# Intramolecular Dynamics within the N-Cap-SH3-SH2 Regulatory Unit of the c-Abl Tyrosine Kinase Reveal Targeting to the Cellular Membrane*[Fn FN3]^♦^

**DOI:** 10.1074/jbc.M113.500926

**Published:** 2013-08-08

**Authors:** Guilherme A. P. de Oliveira, Elen G. Pereira, Giulia D. S. Ferretti, Ana Paula Valente, Yraima Cordeiro, Jerson L. Silva

**Affiliations:** From the ‡ Programa de Biologia Estrutural, Instituto de Bioquímica Médica, Instituto Nacional de Biologia Estrutural e Bioimagem, Centro Nacional de Ressonância Magnética Nuclear Jiri Jonas, Universidade Federal do Rio de Janeiro, 21941-902 Rio de Janeiro, RJ and; the § Faculdade de Farmácia, Universidade Federal do Rio de Janeiro, 21941-902 Rio de Janeiro, RJ, Brazil

**Keywords:** NMR, Protein Dynamics, Protein Structure, Tyrosine Protein Kinase (Tyrosine Kinase), X-ray Scattering, SAXS, c-Abl Protein, Chronic Myeloid Leukemia, Myristoylation

## Abstract

c-Abl is a key regulator of cell signaling and is under strict control via intramolecular interactions. In this study, we address changes in the intramolecular dynamics coupling within the c-Abl regulatory unit by presenting its N-terminal segment (N-Cap) with an alternative function in the cell as c-Abl becomes activated. Using small angle x-ray scattering, nuclear magnetic resonance, and confocal microscopy, we demonstrate that the N-Cap and the Src homology (SH) 3 domain acquire μs-ms motions upon N-Cap association with the SH2-L domain, revealing a stabilizing synergy between these segments. The N-Cap-myristoyl tether likely triggers the protein to anchor to the membrane because of these flip-flop dynamics, which occur in the μs-ms time range. This segment not only presents the myristate during c-Abl inhibition but may also trigger protein localization inside the cell in a functional and stability-dependent mechanism that is lost in Bcr-Abl^+^ cells, which underlie chronic myeloid leukemia. This loss of intramolecular dynamics and binding to the cellular membrane is a potential therapeutic target.

## Teaser

**Background:** c-Abl regulates cell signaling and participates in leukemia pathogenesis via Bcr-Abl chimeric protein.

**Results:** N-Cap and SH3 residues acquire μs-ms motions within the regulatory unit and membrane anchoring upon protein activation.

**Conclusion:** N-Cap-myristoyl tether triggers c-Abl to anchor membrane because of μs-ms dynamics within this regulatory region.

**Significance:** Binding to the membrane is lost in Bcr-Abl chimeric protein, which underlies leukemia.

## Introduction

Receptor tyrosine kinases and nonreceptor tyrosine kinases are the two major groups that compose the kinase superfamily. Within nonreceptor tyrosine kinases, the c-Src kinase belongs to a prominent family known as the c-Src family kinases. The nine c-Src family kinase members, c-Src, Yes, Fyn, Hck, Lck, Lyn, Blk, Fgr, and Yrk, may respond to extracellular stimuli through modular domains, such as the Src homology 3 (SH3)^2^ and the Src homology 2 (SH2) domains, and lipid modifications, such as myristate, for appropriate subcellular localization (1). These proteins share a similar domain arrangement consisting of an SH3, SH2, kinase (SH1), and a membrane-targeting SH4 region at the N terminus, (2) also known as the N-Cap region in c-Abl. The ubiquitously expressed c-Abl protein belongs to the nonreceptor tyrosine kinases (3) and is a key regulator of cell signaling. c-Abl is directly involved in chronic myelogenous leukemia, a human malignancy genetically hallmarked by a reciprocal translocation between the *bcr* and *c-abl* genes (4, 5). The discovery of an abnormal, small chromosome named the “Philadelphia chromosome,” which harbors the *bcr-abl* hybrid gene, was the first consistent chromosomal abnormality associated with a specific type of leukemia and was considered a breakthrough in cancer biology at the time (6).

Because c-Abl activity plays a critical role in signal transduction, the protein is regulated by an autoinhibitory mechanism (7) that involves shifting from an open to a closed conformation (8). The segment of the c-Abl protein (1–260) that includes the N-Cap (∼80 residues), the SH3 and SH2 domains, the SH3-SH2 connector, and the SH2-kinase linker comprises the N-terminal regulatory unit (7, 9, 10). The c-Abl regulatory mechanisms involve post-translational modifications that act as switches, *e.g.* intermolecular phosphorylation/dephosphorylation of Tyr-412 and Tyr-245 (11) and G2-myristate association/dissociation with/from the C-terminal cleft of the kinase domain via the N-Cap segment (10). Additionally, the docking of the SH3 domain to the polyproline region of the SH2-kinase linker (11) and the docking of the SH2 domain to the back of the kinase domain (10) ultimately contribute to the quiescent snapped-lock conformation of the protein. In c-Src, a phosphotyrosine residue in the C terminus of the kinase (Tyr(P)-527) interacts with the SH2 domain and may serve as a surrogate mechanism of the N-Cap-myristoyl switch. Controversially, data from crystallographic studies on c-Src identified a similar pocket for myristate binding (12). More recently, myristoylation was shown to have a positive effect on c-Src kinase activity (13). During the formation of *bcr-abl*, the N-Cap region is lost, (5) resulting in a chimeric protein that is highly active and transforms the cell.

Despite the initial remarkable clinical success of imatinib mesylate (formerly STI-571, Gleevec, Novartis), (14) the efficacy of this drug has gradually decreased (15). In recent years, a new generation of tyrosine-kinase inhibitors has been developed that targets Bcr-Abl moieties in regions other than the kinase catalytic site (16, 17). Recently, an elegant, well designed study demonstrated that the SH2-kinase interaction interface in the Bcr-Abl chimeric protein represents an allosteric target for therapeutic intervention (18).

The SH3 and SH2 domains play a pivotal role as interactive modules in cell signaling, and therefore, understanding the intramolecular dynamics of these domains may offer a platform for compound design with implications for the control of chronic myelogenous leukemia. Because current methods of structure determination have limited applicability to assess the dynamic behavior of macromolecules in their native milieu, we investigated the dynamics of the entire c-Abl regulatory unit (1–260) using an ensemble of biochemical, structural, and cellular approaches. The results presented here demonstrate that the N-Cap and the SH3 domain are coupled via the acquisition of μs-ms timescale motions in the SH3 ligand-binding site and in some residues of the N-Cap once this N-terminal segment binds to the SH2-L region. *In vitro* experiments using the wild-type myristoylated variant (Abl-1b), mutated forms (Abl-G2A/PP and Abl-ΔKD), and the nonmyristoylated wild-type variant (Abl-1a) indicated that the N-Cap-myristoyl tether targets the c-Abl-1b variant to the membrane as an additional mechanism to stabilize this flexible N-terminal region, and this anchoring may represent an early apoptotic signaling event that is lost during the formation of Bcr-Abl.

## EXPERIMENTAL PROCEDURES

### 

#### 

##### DNA Constructs

N-SH3, SH2-L, SH3-SH2, and ABL-RD DNA segments were initially amplified from mononuclear cell aspirates by reverse transcription-polymerase chain reaction (RT-PCR). Primers carrying BamHI/EcoRI sites were used for each construct and subcloned in pGEX-4T1 vector (GE Healthcare) to produce N-terminally GST-bound constructs.

Full-length constructs (Abl-1b wild type, Abl-1a wild type, Abl-G2A, Abl-PP, Abl-G2A/PP, and Abl-ΔKD) were engineered (GenScript Corp.) in pUC57 vector and subcloned in pEGFP-N1 (for Abl-1b isoforms) and pDsRed-Monomer-C1 (for Abl-1a isoform) (Clontech) using XhoI/BamHI sites to produce C-terminally bound GFP- or N-terminally bound DsRed-fused proteins. The mutants G2A, P242E/P249E (PP), G2A/PP, and ΔKD were engineered in the wild-type Abl-1b sequence. All constructs and mutations were confirmed by sequencing.

##### Protein Expression and Purification

For size exclusion chromatography (SEC) and SAXS experiments, proteins were overexpressed in *Escherichia coli* strain BL21(DE3) (Invitrogen) as GST fusion proteins with 1 mm isopropyl-1-thio-β-d-galactopyranoside for 6 h at 37 °C. For a typical protein preparation, 1 liter of cell suspension was thawed, resuspended in saline buffer, lysed by sonication, and centrifuged at 18,000 *g* for 20 min. Supernatant was filtered (0.22 μm) and loaded into a GSTrap (GE Healthcare) equilibrated in saline buffer. Protein was eluted with 50 mm Tris-Cl, 10 mm reduced glutathione, followed or not by GST separation with thrombin (∼10 μg/liter of culture). The GST-N-SH3 and GST-ABL-RD constructs were treated for 2 h with thrombin followed by SEC and immediately frozen at −80 °C until the SAXS measurements. After GST cleavage, proteins were pooled and applied to a Superdex 75 10/300 ultra-fast liquid chromatography column equilibrated with 20 mm phosphate buffer containing 80 mm NaCl, pH 8.0 (buffer A). For the NMR assignment of the linker region, the ^15^N-, ^13^C-labeled SH2-L construct was prepared as described previously (19). For nuclear spin relaxation NMR experiments, ^15^N-labeled constructs were isotopically labeled. NMR samples were prepared with a concentration of 0.1–0.3 mm in buffer A and 10% D_2_O.

##### Size Exclusion Chromatography

SEC was performed using a Shimadzu system ultra-fast liquid chromatography column using a prepacked Superdex 75 10/300 column (GE Healthcare). The running buffer was 20 mm phosphate buffer containing 80 mm NaCl (pH 8.0). A flow rate of 0.7 ml/min was used, and samples (250 μg) were recorded at 280 nm. For the calibration fit, the standard proteins bovine serum albumin (∼66 kDa), carbonic anhydrase (∼29 kDa), RNase A (∼13 kDa), and aprotinin (∼7 kDa), purchased from Sigma, were used. Recombinant proteins used in the calibration were glutathione *S*-transferase dimer (∼52 kDa) from *Schistosoma japonicum*, necrosis- and ethylene-inducing protein 2 (Nep2) monomer (∼24 kDa) and dimer (∼48 kDa) from *Moniliophthora perniciosa*, and the EF-hand troponin C subunit (∼18 kDa) of the troponin complex from *Gallus gallus*. Vitamin B_12_ (∼1.3 kDa) was used as a low molecular weight standard.

##### Spectroscopy Measurements

Fluorescence emission measurements were recorded on an ISSK2 spectrofluorometer (ISS Inc., Champaign, IL). Trp residues were excited at 280 nm, and the emission was recorded from 300 to 400 nm. The protein concentration was 5 μm for all constructs, and the emission intensity was corrected for the contribution of the buffer (20 mm phosphate buffer containing 80 mm NaCl, pH 8.0). Circular dichroism measurements were carried out on a JASCO spectropolarimeter (model J-715). The far-ultraviolet spectra were measured at wavelengths ranging from 190 to 260 nm at 25 °C, resulting in an average of four spectra with a scanning speed of 50 nm/min and a resolution of 0.2 nm for each step. Protein concentration was 126 μm for the N-SH3 construct, 160 μm for the SH2-L, 70 μm for the ABL-RD, and 150 μm for the SH3-SH2. The raw ellipticities (θ) were recorded in a 0.02-cm path length circular cuvette, corrected for the base-line contribution of the buffer (20 mm phosphate buffer containing 80 mm NaCl, pH 8.0), and shown as mean residue ellipticity (MRE) as follows

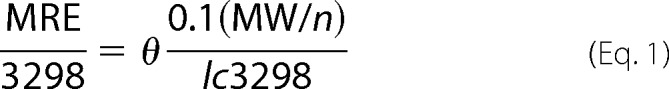
 where MW is the molecular mass (Da), *n* is the number of residues, *l* is the path length, and *c* is the concentration used in mg/ml.

##### Mass Spectrometry

The ABL-RD and N-SH3 construct and the N-SH3 fragments were excised from the time-dependent proteolytic SDS-PAGE and submitted for peptide identification. Tryptic peptides and MS identification were conducted as described previously (20).

##### Scattering Data Collection and Analysis

SAXS data were collected using the SAXS2 small-angle scattering beamline of the National Synchrotron Light Laboratory, Campinas, Brazil, using a two-dimensional position-sensitive detector, with a mica sample holder and a wavelength of 0.155 nm at 20 °C. Data acquisition was performed by taking three frames of 300 s of each sample. The modulus of scattering vector *q* was calculated according to the equation *q* = (4π/λ) sin θ, where λ is the wavelength used, and 2θ is the scattering angle. Data were subtracted from the buffer contribution (20 mm phosphate containing 80 mm NaCl at pH 8.0) and fitted using GNOM (21).

For the N-SH3 construct, data were measured at 1, 2, and 3.4 mg/ml. For the SH2-L construct, data were acquired at 3.5 and 5.5 mg/ml. For the ABL-RD, data were collected at 2.5 and 3 mg/ml, and for SH3-SH2 construct, data were collected at 2.3 mg/ml. Because the value of the radius of gyration (*R_g_*) from Guinier analysis did not change with tested concentrations, the highest concentration in these cases was taken for further analysis. The sample-detector distance was set at 974.09 mm for the SH3-SH2 construct, allowing detection of a *q* in a range from 0.151 to 3.46 nm^−1^. For N-SH3, SH2-L, and ABL-RD, the distance was set at 955.31 mm, and the collected *q* range was from 0.149 to 3.51 nm^−1^. For GST-bound constructs, concentrations used for data collection were 4 mg/ml for the GST-ABL-RD, 14.5 mg/ml for the GST-N-SH3 construct, and 9.5 mg/ml for the GST-SH3-SH2. For these constructs, distance was set at 973.68 mm, and a *q* range from 0.151 to 3.28 nm^−1^ was taken. Because quality of the SAXS data depends on monodispersity of molecules and no aggregation, samples were centrifuged at 10,000 *g*, 10 min, 20 °C immediately before data acquisition.

*R_g_* and *D*_max_ were determined by running the GNOM software, in which a range of input distances is tested to best fit the experimental data and the *R_g_* estimation is obtained by Guinier analysis. Fourier transformation of the scattered data results in a distribution of the interatomic distance (*P*(*r*)) contained in the molecules, which allows us to graphically display peculiarities of the particle shape.

##### Shape Restoration from the SAXS Data

Low resolution shape restorations were performed for all constructs with 10 independent calculation trials using the GASBOR program (22) and averaged in DAMAVER (23). The precision of the averaged envelope was available from the normalized spatial discrepancy parameter, and a reasonable agreement between individual models was achieved with low values of normalized spatial discrepancy parameters (∼1.0). SUPCOMB (24) or manual adjust was used to superimpose the shapes with the crystal structures 1OPK (c-Abl core), 2ABL (SH3-SH2), and 1UA5 (GST).

##### Resonance Assignment and Nuclear Spin Relaxation Experiments

NMR data were acquired at 25 °C using a Bruker Avance III 800 MHz. A set of three-dimensional triple-resonance spectra (HSQC, HNCA, HNCACB, HNCO, HN(CA)CO, and CBCA(CO)NH) was collected for the SH2-L construct for backbone resonance assignment of the SH2-kinase linker region. A prevalence of overlapping cross-peaks prevented the backbone assignment of the N-Cap segment in the ABL-RD construct. The SH3 and SH2 triple-resonance assignments from the Biological Magnetic Resonance Data Bank (BMRB; BMRB accession number 4251) were used to aid in the assignment process of the SH3 and SH2 domains.

For relaxation experiments, two relaxation parameters were measured: the longitudinal relaxation rate *R*_1_ and the transverse relaxation rate *R*_2_. Measurements were collected for the SH2-L, N-SH3, and ABL-RD constructs. *R*_1_ and *R*_2_ report on changes of the diffusion properties of the protein. A decrease in *R*_2_/*R*_1_ values indicates thermal flexibility motions, which are typically observed for loop regions on the picosecond to nanosecond timescale. Additionally, an increase in the values of *R*_2_/*R*_1_ reflects contributions for conformation exchange motions on the microsecond to millisecond timescale.^15^N *R*_1_ values were measured from two-dimensional HSQC spectra with relaxation delays 20, 50, 100, 200, 250, 500, 750, 1000, and 1500 ms for the SH2-L and N-SH3 construct and 50, 100, 200, 500, 700, 1000, 1500, and 2000 ms for the ABL-RD construct; ^15^N *R*_2_ values were measured with relaxation delays 16, 48, 80, 112, 144, 176, 208, 240, 272, and 304 ms for the SH2-L and N-SH3 constructs and 16, 32, 48, 64, 80, 96, 112, 128, and 144 ms for the ABL-RD construct. Data points were fitted for the length of the parametric relaxation delay as an exponential decay function as follows: *I*(*t*) = *e*^−^*^Rt^*, where *I* is the intensity of the magnetization.

##### Transfection and Immunoblots

HEK293 or VERO cells were cultured in Dulbecco's modified Eagle's medium supplemented with 10% fetal bovine serum. Cells were transfected with pEGFP-N1 or pDsRed-C1 vectors containing the full-length c-Abl constructs using the calcium phosphate protocol (for HEK293 cells) or the Lipofectamine protocol (for VERO cells). Either 15 h or 48 h after transfection, cells cultured on coverslips were prepared for microscopy. For immunoblots, HEK293 cells (48 h after transfection) were lysed with 5 mm Tris-HCl, pH 7.4, 10 mm EDTA, 1 mm Na_3_VO_4_, 5 mm NaF, 1 μm okadaic acid, 1× protease mixture inhibitors (Sigma), and 1 mm PMSF and centrifuged at 20,000 × *g* for 20 min at 4 °C. To assess the autophosphorylation profile of c-Abl full-length constructs and apoptotic markers, transiently transfected HEK293 cells were immunoblotted with specific antibodies against Tyr(P)-245 (Sigma, C5365) and Tyr(P)-412 (Sigma, C5240), c-Abl protein, p53 DO-1 (Santa Cruz Biotechnology, sc-126), and Bax (Calbiochem, PC66) using the Odyssey system (LI-COR).

##### Fluorescence Microscopy

Following the transfection protocol, cells cultured on coverslips were washed twice with phosphate buffer and fixed. Cells were counterstained with Image-IT plasma membrane and nuclear labeling kit (Molecular Probes, I34406) and imaged using an LSM 510 confocal microscope (Zeiss).

## RESULTS

### 

#### 

##### Small Angle X-ray Scattering Analysis of ABL-RD Constructs

**FIGURE 1.**
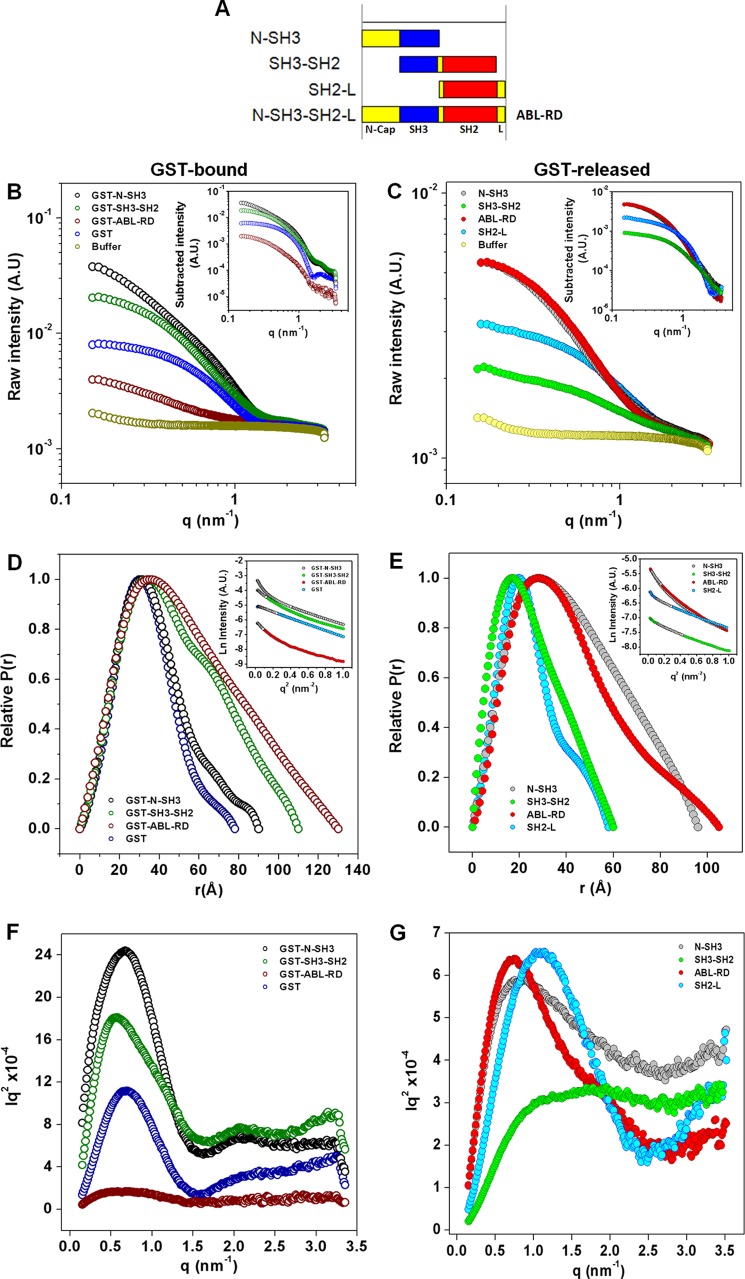
**SAXS scattering data.** *A*, constructs from the c-Abl regulatory unit. *A. U.*, arbitrary units. *B* and *C*, raw scattered intensity *versus q* for the GST fusion and GST-released c-Abl regulatory domain constructs, respectively. *Insets* show data from which the buffer contribution has been subtracted. *D* and *E*, plots of the interatomic distances (*P*(*r*) function). *Insets* show the Guinier plots for the GST-bound and GST-released c-Abl regulatory domain constructs. The linear regions of the low *q* section are shown for each construct. Data used for the *R_g_* estimation are highlighted as *open circles* and were calculated via the angular coefficient (α) of the linear regression of these data as follows: α = −*R_g_*^2^/3. *F* and *G*, Kratky plots. Plots are shown in the identical schematic color as depicted in the specific legend for each plot.

To evaluate changes in the orientation of the N-Cap within the entire c-Abl regulatory unit (ABL-RD), we performed SAXS analysis on GST fusion and GST-free constructs (Fig. 1*A*): 1) SH2-L, 2) GST, 3) GST-N-SH3 and N-SH3, 4) GST-ABL-RD and ABL-RD, and 5) SH3-SH2. Raw data are depicted in Fig. 1, *B* and *C*. The *R_g_* values were calculated from the Guinier analysis of the low *q* region of scattered plots (Fig. 1, *D* and *E*, *insets*) and from the *P*(*r*) distribution of the interatomic vectors (Fig. 1, *D* and *E*) and are summarized in Table 1. The measured values of *R_g_* and *D*_max_ for the N-SH3 and ABL-RD constructs (32.9/96 Å and 32.4/105 Å, respectively) revealed an elongated particle when compared with the values obtained for the SH2-L and SH3-SH2 constructs (19.1/58 Å and 19.4/60 Å, respectively), presumably because of the presence of the N-Cap segment. The similarity of the *R_g_* value in the shorter N-SH3 construct when compared with the ABL-RD construct may be reflected by the occupational volume adopted by the N-Cap region in the N-SH3 construct.

**TABLE 1:** **Structural parameters obtained from SAXS**

Sample	*R*_*g* Guinier_	*R*_*g* Gnom_	*D*_max_ _Gnom_	NSD*^a^*
	Å	Å	Å	
SH2-L	20.5	19.1	58	0.824 ± 0.017
GST	25.8	25.6	78	1.034 ± 0.023
GST-N-SH3	28.7	28.8	90	
N-SH3	35.2	32.9	96	1.791 ± 0.057
SH3-SH2	19.2	19.4	60	0.688 ± 0.01
GST-ABL-RD	43.7	42.2	130	1.638 ± 0.163
ABL-RD	33.5	32.4	105	1.529 ± 0.057

*^a^ N*ormalized spatial discrepancy obtained from DAMAVER.

Comparisons of the *P*(*r*) function among the constructs (Fig. 1, *D* and *E*) reveal that the N-SH3 construct demonstrates a bell shape, as expected for a spherically shaped molecule. This result presumably indicates that the N-Cap region in the N-SH3 construct assumes heterogeneous conformations in solution when compared with the other constructs. Controversially, the *P*(*r*) distribution for the GST fusion N-SH3 construct was similar to that obtained for the GST protein alone, which indicates that the N-Cap in the GST fusion protein assumes a compact orientation. Furthermore, for the SH3-SH2 construct, a symmetrically distributed *P*(*r*) function was obtained, which is expected for a prolate structure, evidence that this bimodular domain may assume a compact conformation (Fig. 1*E*). In contrast, a more skewed pattern for ABL-RD was observed, which suggests an elongated structure.

To evaluate the degree of folding, the Kratky plot can be useful (25). The Kratky distribution for the GST fusion proteins was similar for all constructs and indicative of folded proteins (Fig. 1*F*). However, analysis of the Kratky plots for the GST-free constructs (Fig. 1*G*) indicated that N-SH3 exists in a partially unfolded conformation when compared with the ABL-RD and SH2-L constructs because it demonstrates a continuous increase in I*q*^2^ with *q*. Note that the Kratky plot for the SH3-SH2 construct also has some attributes of unfolding; however, the Kratky shape for modular multidomain proteins with flexible linkers particularly reveals a mixture of features for folded and unfolded proteins similar to the features obtained for a partially unfolded state (25, 26).

##### ABL-RD Shape Reconstructions from SAXS

**FIGURE 2.**
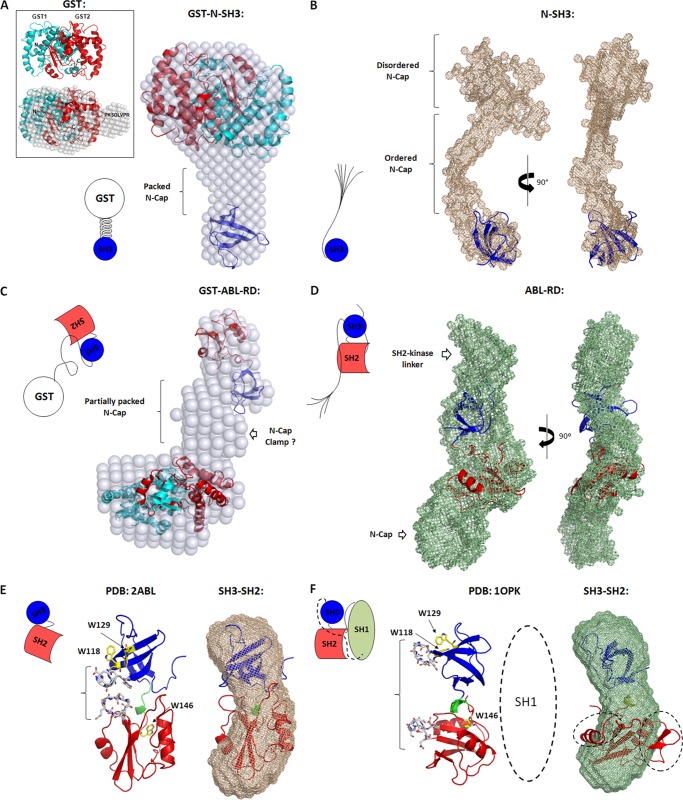
**N-Cap organization as determined by SAXS.** *A*, GST-N-SH3 construct. The GST crystal structure (*cyan* and *red*) from PDB entry 1UA5 and the SH3 domain from the c-Abl crystal structure PDB entry 1OPK were superimposed on the molecular envelope (*spheres*). *Inset* shows shape reconstruction for the GST protein. The additional volume in the GST envelope unaccounted for in the GST crystal structure presumably refers to the thrombin recognition site (PKSDLVPR) left at the C terminus. *B*, the GST-released N-SH3 construct. *Blue*, superposition of the SH3 domain. The shape of N-SH3 is shown as *mesh*. Note the heterogeneous orientation adopted by the N-terminal region of the N-Cap in this construct. *C*, the GST-ABL-RD construct. The GST dimer and the SH3-SH2 domains (*blue* and *red*, respectively) were superimposed on the molecular envelope (*spheres*). In the region assumed to be occupied by the partially compact N-Cap, note the clamp organization for the N-Cap region in this construct. *D*, the GST-released ABL-RD construct. The SH3-SH2 domains were superimposed onto the shape (*mesh*). Note the unaccounted volume occupied by the N-Cap and the SH2-kinase linker. *E*, shape reconstruction for the SH3-SH2 construct using SAXS. Ribbon representation of the SH3-SH2 crystal structure (PDB entry: 2ABL) is shown. Interactions between the SH3 and SH2 domain and Trp residues are highlighted in *gray* and *yellow sticks*, respectively. The SH3 domain, SH2 domain, and SH3-SH2 connector are shown in *blue*, *red*, and *green*, respectively. *F*, ribbon representation for the SH3-SH2 crystal structure (PDB entry: 1OPK). Contacts that were lost between the SH3 and the SH2 domains, and Trp residues are highlighted in *gray* and *yellow sticks*, respectively. SH3, SH2, and SH3-SH2 connector are colored as in *panel E*. The SH2-kinase linker and the kinase domain were depleted for visual inspection. Shape reconstruction for the SH3-SH2 construct was superimposed to the corresponding region of the 1OPK crystal structure. Unmatched regions of the crystal into the SH3-SH2 envelope were highlighted (*dashed lines*). Schematics for each shape reconstruction were built to illustrate the data.

The consensus shape reconstructions for the SH2-L domain (data not shown) and the GST protein (Fig. 2*A*, *inset*) were consistent with the corresponding crystal structures. The GST protein naturally formed dimeric species, as observed for the superimposed structures (Fig. 2*A*, *inset*).

The GST dimer in the GST fusion N-SH3 construct revealed a close proximity to the SH3 domain. Because the N-Cap segment is flanked by these domains, this result confirms that the N-Cap assumes a compact orientation (Fig. 2*A*) in the absence of the SH2-L domain and upon N-terminal rigidity of the N-Cap (GST fusion). Remarkably, the consensus shape reconstruction for the GST-free N-SH3 construct revealed that the N-Cap segment existed in two different orientations in solution (Fig. 2*B*): an ordered region located in the initial two-thirds of the SH3 domain and a disordered region located in one-third of the N terminus that presumably reflects the heterogeneity of the conformations.

Shape restoration for the GST fusion ABL-RD construct revealed a partially compact N-Cap segment, likely resulting from the influence of the SH2-L region (Fig. 2*C*). Moreover, an additional volume was observed in the density region assumed to be occupied by the N-Cap segment, which would be consistent with a clamp inside the N-Cap (Fig. 2*C*). The GST-free ABL-RD molecular envelope indicates additional volume unaccounted for in the crystal structure (Fig. 2*D*); however, in this case, this additional volume, which presumably corresponds to the N-Cap and SH2-kinase linker segments, was present in an ordered orientation and very near to the SH3-SH2 globular domains. Shape restoration of the SH3-SH2 construct revealed a best superposition fit with the crystal structure of only the SH3-SH2 domains (Protein Data Bank (PDB) entry: 2ABL and Fig. 2*E*) rather than the structure of the SH3 and SH2 domains within the entire c-Abl core (PDB entry: 1OPK and Fig. 2*F*).

##### Characterization of the Entire N-Cap in the c-Abl Regulatory Unit (ABL-RD)

**FIGURE 3.**
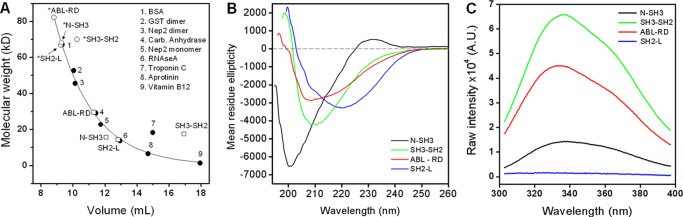
**Spectroscopic peculiarities from the N-Cap within the c-Abl regulatory unit.** *A*, SEC calibration curve for recombinant ABL-RD constructs. The exponential decay fit was performed with the standard proteins. The GST fusion constructs (marked with an *asterisk*) are shown as *open circles*, the GST-free constructs are shown as *open squares*, and standard proteins are shown as *filled circles. Carb. Anhydrase*, carbonic anhydrase. *B*, far-ultraviolet circular dichroism spectra of GST-released constructs of the ABL-RD expressed as mean residue ellipticity. *C*, fluorescence spectroscopic measurement of the fluorescence emission of Trp residues (Trp-118 and Trp-129) in the SH3 domain. *A. U.*, arbitrary units.

A crystallographic and mutagenic study pinpointed that residues 15–56 of the N-Cap play no role in the autoregulation of c-Abl and thus were deleted from the N-Cap region (9). However, residues spanning from 2–14 and 61–82 are important but not yet fully understood for the myristoyl switch (9). To further obtain insights into the intramolecular contributions among the entire N-Cap region (1–83) and the SH3, SH2, and SH2-kinase linker regulatory domains, we conducted SEC of the ABL-RD unit (1–260) and of the shorter constructs (SH2-L: 137–260; N-SH3: 1–138; SH3-SH2: 84–239). The retention volumes of the ABL-RD and shorter constructs were consistent with the expected values as fitted for standard proteins (Fig. 3*A*, *filled circles*), except for the SH3-SH2 domain, which demonstrated an anomalous elution when compared with the others (Fig. 3*A*, *open squares*). The orientation of the globular domains in SH3-SH2 construct may allow the formation of a compact rearrangement between them, probably because of the conformational flexibility assumed for the SH3-SH2 connector that facilitates SH3-SH2 contacts or because of the anisotropic tumbling of the molecule. Troponin C is formed by two globular domains connected by an exposed α-helix and, similar to the SH3-SH2 construct, did not elute at the expected volume according to the SEC calibration curve (see *number 7* in Fig. 3*A*). Similar to the SH3-SH2 construct, the GST-SH3-SH2 (see *SH3-SH2 in Fig. 3*A*) did not elute at the expected retention volume for its molecular weight. These data indicate that bimodular proteins connected by flexible regions, as observed for SH3 and SH2 domains, may rearrange their orientation in solution and that the presence of the entire N-Cap segment specifically prevents this coupling.

Secondary structures for the different constructs assessed by circular dichroism (Fig. 3*B*) are consistent with the profile expected from c-Abl crystal structures, but the CD spectrum for the N-SH3 construct was in line with the lack of secondary structure in the most part of the N-Cap segment. However, tertiary changes indicated by the Trp fluorescence reveal that Trp-118 and Trp-129 in the SH3 domain are well quenched in the sole presence of the N-Cap segment when compared with the other constructs (Fig. 3*C*). The average energy values (center of spectral mass, cm^−1^) of the Trp-118 and Trp-129 emission for the ABL-RD, SH3-SH2, and N-SH3 constructs were 29,056, 28,979, and 28,870 cm^−1^, respectively. The lower value obtained for the N-SH3 construct suggests that Trp-118 and Trp-129 are more exposed to the solvent in this construct than in the SH3-SH2 and ABL-RD constructs. Inclusion of the SH2-L region with the N-SH3 domains (*i.e.* the ABL-RD construct) resulted in a fluorescence intensity of Trp-118 and Trp-129 that was three times greater than that of the N-SH3 construct (Fig. 3*C*), indicating that Trp-118 and Trp-129 now sample an environment in which some internal quenching is reduced. The fluorescence contribution from Trp-146 in the SH2-L domain does not account for this increased fluorescence (Fig. 3*C*). Additionally, these Trp residues are less exposed to the solvent in the ABL-RD construct when compared with the N-SH3 construct. The deletion of the N-Cap and SH2-kinase linker region from the ABL-RD construct (*i.e.* the SH3-SH2 construct) resulted in an ∼5-fold increase in the Trp fluorescence when compared with the N-SH3 construct (Fig. 3*C*). However, the variation of the average energy of the Trp emission when comparing the ABL-RD and SH3-SH2 construct (Δυ = 77 cm^−1^) with the ABL-RD and N-SH3 construct (Δυ = 186 cm^−1^) indicates that the SH2-L region influences the maintenance of the SH3 tertiary structure to a greater degree than the flexible regions (both the N-Cap and the SH2-kinase linker). These spectroscopy results provide peculiarities on the structural behavior within the c-Abl regulatory unit and indicate that these constructs maintain their secondary and tertiary interactions.

**FIGURE 4.**
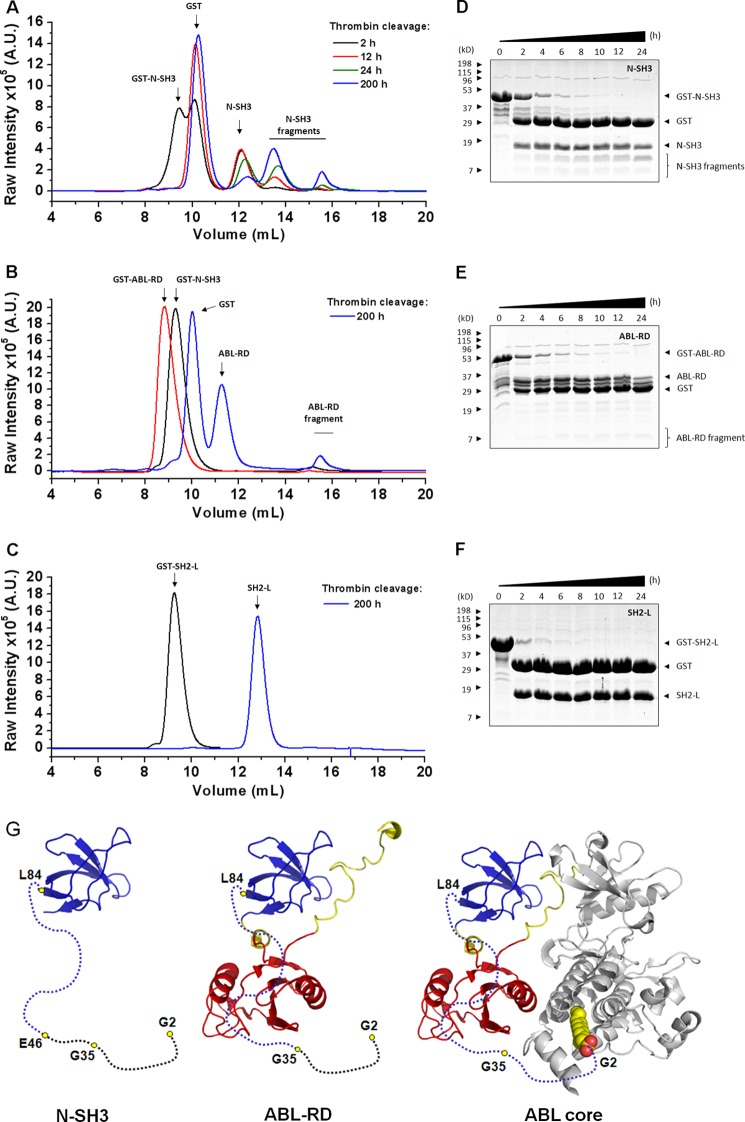
**Mechanistic model for c-Abl inhibition.** *A–C*, temporal SEC analysis for the GST-bound and GST-released N-SH3, ABL-RD, and SH2-L constructs, respectively. *A. U.*, arbitrary units. *D–F*, 15% SDS-PAGE gels stained with Coomassie Blue G250 show proteolytic fractions at different incubation times for N-SH3, ABL-RD, and SH2-L constructs, respectively. Coomassie Blue G250 staining was recorded using the Odyssey system (LI-COR). *G*, proposed model for the stability of N-Cap. *Left*, N-Cap proteolysis in the N-SH3 construct; *middle*, N-Cap proteolysis in the ABL-RD; and *right*, additional stability for the N terminus of the N-Cap (from Gly-2 to Gly-35) would be provided by myristate (*sphere representation*) binding. *Black dots* represent cleaved segments.

To assess the propensity for proteolytic cleavage of the N-Cap in the presence and absence of the SH2-L domain, we designed a time-dependent proteolytic assay coupled with MS confirmation. Comparison of the ABL-RD and N-SH3 constructs revealed a different susceptibility for proteolytic cleavage of the N-Cap. The increased labeling of fragments corresponding to the N-Cap sequence was less pronounced in the ABL-RD construct than in the N-SH3 construct, as shown by SEC (Fig. 4, *A–C*), SDS-PAGE analysis (Fig. 4, *D–F*), and peptide identification (supplemental Table S1). The SH3-SH2 connector was previously shown to interact with the shorter N-Cap segment (2–14 and 61–82) through Ser(P)-69, evidence that this N-terminal segment may also act as a stabilizer in addition to presenting the myristoyl modification during kinase inhibition (8). Our data demonstrate that the SH2 domain participates on N-Cap stabilization in a phosphorylation- and kinase-independent model. Sequence identification by MS revealed the region from Gly-36 to the beginning of the SH3 domain as the minimal segment in the N-Cap that may account for this stabilization through interactions with SH2-L in this manner (supplemental Table S1 and Fig. 4*G*). Complementary stability of the corresponding N-Cap region from Gly-35 to Gly-2 might be achieved via the binding of myristate to the C-terminal cleft in the kinase domain. Here we provide a mechanistic interpretation for the c-Abl inhibition through the N-Cap-myristoyl tether. Likely, for those interactions between Ser(P)-69 and the N-Cap in c-Abl obtained from insect cells, the N-Cap-SH2 interactions may participate in guiding myristate binding as a cascade-like mechanism of inhibition. It is reasonable that SH2 contacts and myristate binding are uncoupled but complementary for the stability of the N-Cap, and consequently, kinase inhibition.

##### Targeting Intramolecular Interactions within the ABL-RD

**FIGURE 5.**
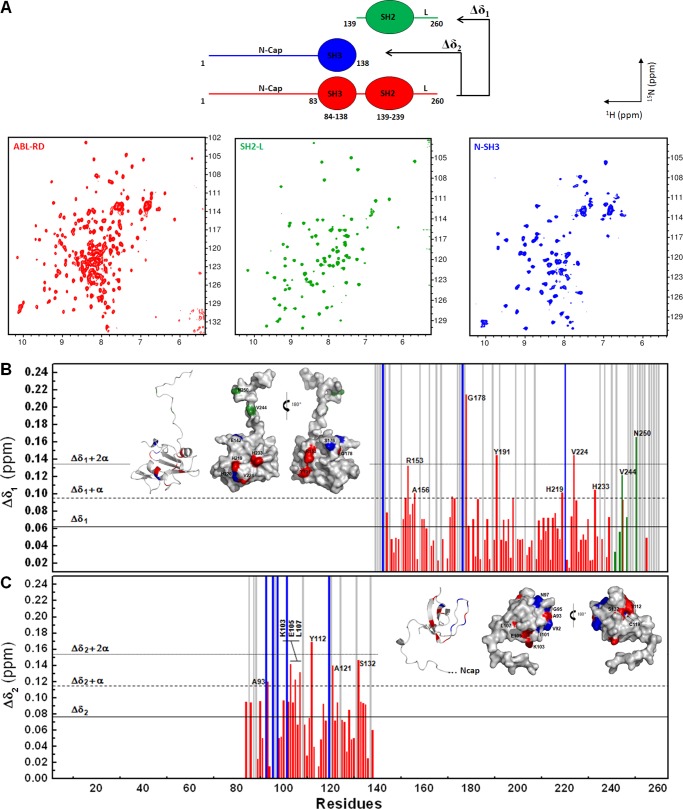
**Targeting intramolecular sites within the c-Abl regulatory unit.** *A*, ^1^H-^15^N HSQC NMR spectra for ABL-RD, SH2-L, and N-SH3 constructs. The cross-peaks are colored using the color code in the schematic diagram. *B* and *C*, ^1^H-^15^N chemical shift perturbation plots comparing the ABL-RD with the SH2-L construct (Δδ_1_) and the N-SH3 construct (Δδ_2_), respectively. The significance of the shifted residues is shown as *horizontal lines*: *solid*, *dashed*, and *dotted lines* indicate the average of the residues, the average plus one standard deviation (α), and the average plus two standard deviations, respectively. Shifted, missing, shifted and decreased in intensity, and unassigned residues are colored in *red*, *blue*, *green*, and *gray*, respectively. *Insets* show ribbon and surface representations of the SH2-L and N-SH3 constructs, respectively. Perturbed residues are highlighted with the same color scheme as that used in the Δδ plots.

We designed chemical shift perturbation (Δδ) analyses of ^1^H-^15^N by NMR HSQC (Fig. 5*A*) spectra to map critical moieties that are affected by intramolecular interactions within the ABL-RD. We performed two Δδ analyses using the entire ABL-RD construct as a template.

Δδ1 revealed the most disturbed residues in the SH2-L domain upon deletion of the N-SH3 region (Fig. 5*B*). We observed that 12 residues in the SH2-L domain underwent significant ^1^H-^15^N backbone perturbations in three different manners: shifts without significant variation of the peak intensity (Arg-153, Ala-156, Gly-178, Tyr-191, His-219, Val-224, and His-233), shifts with reduction in the peak intensity (Val-244 and Asn-250), and the disappearance of the peak (Glu-142, His-220, and Ser-176). Mapping these residues in the SH2-L crystal structure revealed two clusters that represent potential sites for the interaction of the SH2-L with the N-Cap (His-219, His-220, Val-224, and His-233) and of the SH2-L with the SH3 domain (Glu-142, Ser-176, and Gly-178) (Fig. 5*B*, *inset*). Additionally, deletion of the SH3 domain revealed that residues Val-244 and Asn-250 in the SH2-kinase linker region may sense the binding of the SH3 domain to the polyproline site composed of Pro-242 and Pro-249.

Similarly, the effect of SH2-L deletion on the SH3 domain was shown by Δδ2 analyses (Fig. 5*C*). The most disturbed sites were clustered in the long loop region between the β1-β2 strands (Val-92, Ala-93, Gly-95, Asn-97, Ile-101, and Lys-103), demonstrating two different perturbation profiles for the region where the SH2-kinase linker segment interacts with the SH3 domain in the SH3 crystal structure (Fig. 5*C*, *inset*).

##### Dynamic Properties of the ABL-RD

**FIGURE 6.**
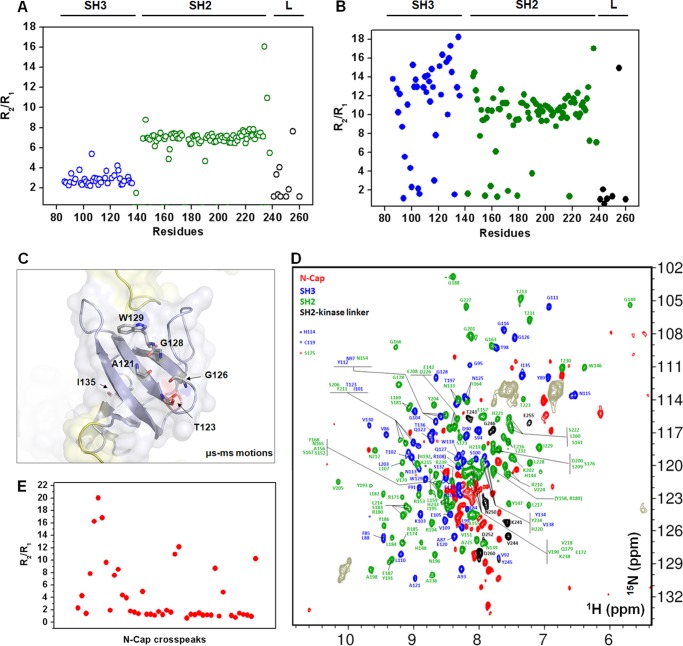
**The N-Cap and SH3 domain acquire slow contribution motions.** *A*, *R*_2_/*R*_1_ plot *versus* the residues for the short constructs N-SH3 (*blue*) and SH2-L (*green* and *black*). Assigned residues from the SH2-kinase linker (*L*) in the SH2-L construct are shown in *black. B*, *R*_2_/*R*_1_ plot *versus* the residues for the entire ABL-RD construct. The color scheme is identical to that used in *A. C*, surface representation illustrating the top view of the SH3 domain. Residues involved in μs-ms motions are shown as *sticks*. To map μs-ms motions, *R*_2_/*R*_1_ changes were considered to be significant for values that were at least one calculated standard deviation away from the averaged residues (9.76 ± 4.22). *D*, representative ^1^H-^15^N HSQC NMR spectra from the entire ABL-RD (1–260) construct with labeled cross-peaks. The ^1^H-^15^N coherences are shown for the N-Cap (*red*), SH3 (*blue*), SH2 (*green*), and the SH2-kinase linker (*black*). Side chain signals are colored *brown*. Residues unassigned were: N-Cap (from 1 to 83), Asp-96, Lys-124, Ser-140, Leu-141, Lys-143, Ser-145, Ser-162, Ser-175, Ser-199, Ser-207, Asn-240, Val-247, Ser-248, Tyr-251, Lys-253, Trp-254, Met-256, Glu-257, Arg-258, and Thr-259. *E*, *R*_2_/*R*_1_ plot *versus* the N-Cap cross-peaks indicates that this segment acquires μs-ms motions in the ABL-RD construct.

We investigated the dynamic properties of the ABL-RD and the short isolated constructs N-SH3 and SH2-L using relaxation methodologies. We measured the longitudinal relaxation rate *R*_1_ and the transverse relaxation rate *R*_2_ for the ^1^H-^15^N nuclear spins. The short constructs of the ABL-RD (*i.e.* N-SH3 and SH2-L) revealed a homogeneous distribution of *R*_2_/*R*_1_ among residues (Fig. 6*A*). However, we observed a differential contribution for *R*_2_/*R*_1_ for the entire ABL-RD (Fig. 6*B*). This observation relies on the acquisition of differential internal motion, presumably because of the cross-talk among individual segments of the regulatory unit. Note that we observed μs-ms contributions in some residues of the SH3 domain in the ABL-RD construct; these contributions were not observed in the short N-SH3 construct. Remarkably, this contribution was not observed in the SH2 domain of ABL-RD when compared with the SH2-L construct. Previous dynamic data from the connected SH3-SH2 domains of the c-Abl protein also revealed a homogeneous distribution of *R*_2_/*R*_1_ among residues (27). These observations suggest that the N-Cap and the SH2-kinase linker segments of the protein participate in the acquisition of SH3 μs-ms motions in a SH1-independent manner. Further analysis of the relaxation data demonstrates that most of the residues located in close proximity to the c-Abl SH3-binding site (Ala-121, Thr-123, Gly-126, Gly-128, Trp-129, and Ile-135) are involved on the μs-ms timescale (Fig. 6*C*).

From the ^1^H-^15^N HSQC spectra of the ABL-RD, ∼40 ^1^H-^15^N nuclear spins (50% of the entire N-Cap region) originated from the N-Cap region (Fig. 6*D*). Further evaluation of the *R*_2_/*R*_1_ values from this region revealed that most of the spins experience fast motions (on the ps-ns timescale), as expected for a flexible segment, but slow contribution motions (on the μs-ms timescale) were also observed in this region (Fig. 6*E*). The slow contribution dynamics within the N-Cap segment may reflect the transfer of motion from the N-Cap to the SH3 domain through N-Cap-SH2 binding.

##### Membrane Targeting of the N-Cap-Myristoyl Tether

**FIGURE 7.**
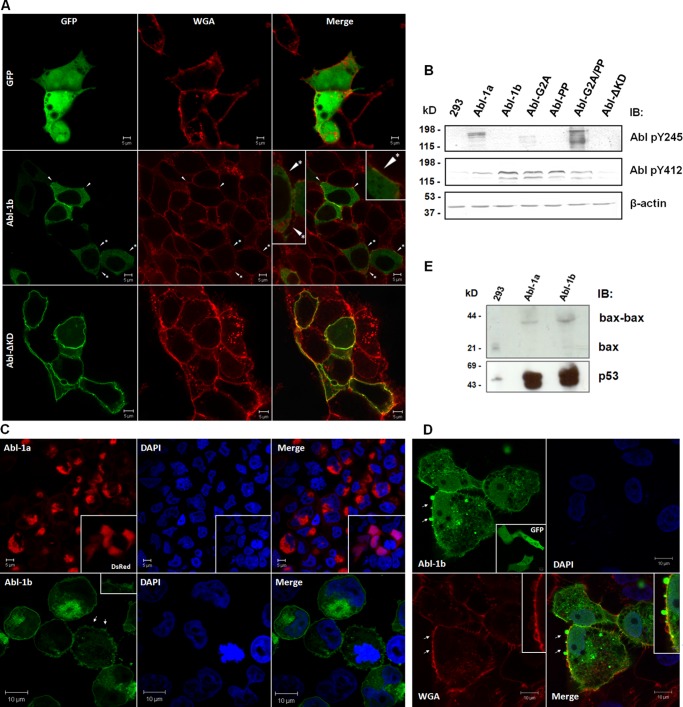
**Additional role for the N-Cap-myristoyl tether.** *A*, HEK293 cells were transiently transfected with the indicated GFP-fused full-length constructs and prepared for confocal microscopy 15 h after transfection (*first column*). The membrane was stained with wheat germ agglutinin (*second column*), which colocalized (*arrowhead*) with the c-Abl constructs (*third column*). Images show 0.9-μm confocal sections in the mid-nuclear planes. Colocalization of the Abl-1b with membrane regions is amplified in the *inset* (*arrowhead* with an *asterisk*). *B*, whole cell lysates from transiently transfected HEK293 cells were prepared and immunoblotted (*IB*) with specific antibodies against c-Abl phosphorylated tyrosine 412 and 245. *C*, HEK293 cells were prepared as in *A* and imaged by confocal microscopy 48 h after transfection to assess the c-Abl distribution in apoptotic cells. *DsRed* indicates the distribution of Abl-1a, and *GFP* indicates the distribution of Abl-1b. *Insets* show DsRed null vector distribution and the blebbing of apoptotic cells. *D*, VERO cells were transiently expressed with the GFP null vector (*inset*) or the Abl-1b protein using the Lipofectamine reagent and prepared for confocal microscopy 48 h after transfection. Apoptotic cells revealed a major distribution of the myristoylated c-Abl isoform into membrane regions. *Arrows* indicate membrane blebbing. *WGA*, wheat germ agglutinin. *E*, as in *B*, cells were immunoblotted with antibodies against the apoptotic markers p53 and Bax.

Our SAXS results indicated that there are at least two different orientations for the entire N-Cap segment in the GST fusion protein in which GST dynamically restricts the N-terminal region of the N-Cap: a compact orientation, as observed for the shape reconstruction from the GST-N-SH3 construct, and a partially compact orientation that is consistent with a clamp orientation for the N-Cap, as observed for the GST-ABL-RD construct. *In vivo*, myristoyl plays an important role in c-Abl regulation via G2-myristate association/dissociation with/from the C-terminal cleft of the kinase domain (9). This regulatory tether is mediated by the N-Cap region. Currently, information regarding the fate of this regulatory tethering upon c-Abl activation remains elusive. We questioned whether the myristate moiety in the N-Cap-myristoyl tether would trigger the c-Abl to anchor to the membrane. Confocal microscopy revealed slight colocalization of the wild-type myristoylated isoform (Abl-1b) with the membrane, and this distribution was enhanced upon kinase domain deletion (Fig. 7*A*). Immunoblots showed that the myristoylated protein (Abl-1b) was extensively phosphorylated solely at Tyr-412, whereas the nonmyristoylated isoform (Abl-1a) was dually phosphorylated at Tyr-245 and Tyr-412 (Fig. 7*B*). Additionally, the Abl-G2A isoform demonstrated minor phosphorylation at Tyr-245. These results indicate that the regulatory role of the N-Cap-myristoyl tether to inhibit the c-Abl kinase by presenting the myristoyl group to the kinase domain is functional (as evidenced by minor phosphorylation at Tyr-245 in the Abl-G2A isoform and no phosphorylation at Tyr-245 in the c-Abl-1b isoform). However, Tyr-412 was phosphorylated in the wild-type and G2A mutant, supporting the hypothesis that the N-Cap-myristoyl tether may regulate the increase of the kinase activity at least by its clamping function, which favors interactions between the SH3 domain and the SH2-kinase linker and may ultimately prevent the phosphorylation of Tyr-245. The phosphorylation of Tyr-412 in these constructs is consistent with the basal activity for the c-Abl kinase (11). Our results indicate that the myristoyl group not only participates in the inhibition of the kinase domain but also targets the active c-Abl kinase (at least activated at Tyr-412) to anchor in the membrane. These results support an accessory role for this c-Abl lipid modification inside the cell.

During our experiments, we serendipitously discovered a possible role for this lipid modification in anchoring the c-Abl kinase to the membrane. The c-Abl kinase participates in several proapoptotic networks, *e.g.* its involvement in the Pin1-p73 phosphorylation-dependent interaction that leads to p73 activation and transcriptional activity upon genotoxic treatment (28, 29). Activation of the c-Abl proapoptotic pathway leads to translocation of the protein to the membrane, as revealed by confocal microscopy of post-transfection experiments (48 h) (Fig. 7, *C* and *D*); this translocation was not observed for the nonmyristoylated form (Abl-1a). Membrane blebbing, p53 overexpression, and Bax dimerization were observed in the apoptotic cells (Fig. 7, *C–E*). This finding supports a possible role for this N-terminal segment to target the c-Abl protein to the membrane during apoptotic signaling. A possible explanation in light of these results would be that the c-Abl kinase is targeted to the membrane during the early apoptotic signaling to phosphorylate specific apoptotic substrates. Further experiments are required to assess the participation of the membrane-bound c-Abl kinase in the apoptotic interactome in this subcellular environment.

## DISCUSSION

In this study, we addressed changes in the intramolecular dynamics within the c-Abl regulatory unit by presenting the N-Cap with an alternative function in the cell as c-Abl becomes activated. We demonstrate that, in addition to the well known function of regulating c-Abl kinase activity, the N-Cap-myristoyl tether may direct the c-Abl protein to anchor in the membrane as an additional mechanism to stabilize this N-terminal segment, which may also be linked to an early apoptotic signaling.

The SH3-SH2 construct is formed by two globular domains connected by a flexible linker (NSLEK). The orientations of the crystal structure of c-Abl (PDB entry: 1OPK; residues 1–531) (10), the crystal structure of only the SH3-SH2 domains (PDB entry: 2ABL; residues 76–237) (30), and a molecular dynamics simulation from a c-Abl triple mutant (S140G,L141G,E142G) within the SH3-SH2 connector (10) support the idea that the SH3-SH2 connector adopts a rigid structure when the SH3 and SH2 domains are connected. The orientation of both domains in the 2ABL crystal structure reveals that they can interact with each other through the SH3-SH2 loop regions (30) (Fig. 2*E*). It is reasonable to conclude that both domains interact with each other to stabilize the bimodular structure and prevent flexibility via the SH3-SH2 connector. The presence of the N-Cap and the SH2-kinase linker segments would restrict the flexibility of the SH3-SH2 connector, providing rigidity to the overall SH3-SH2 unit, as observed in the 1OPK crystal structure in which the SH3-SH2 connecting loops are distant from each other.

**FIGURE 8.**
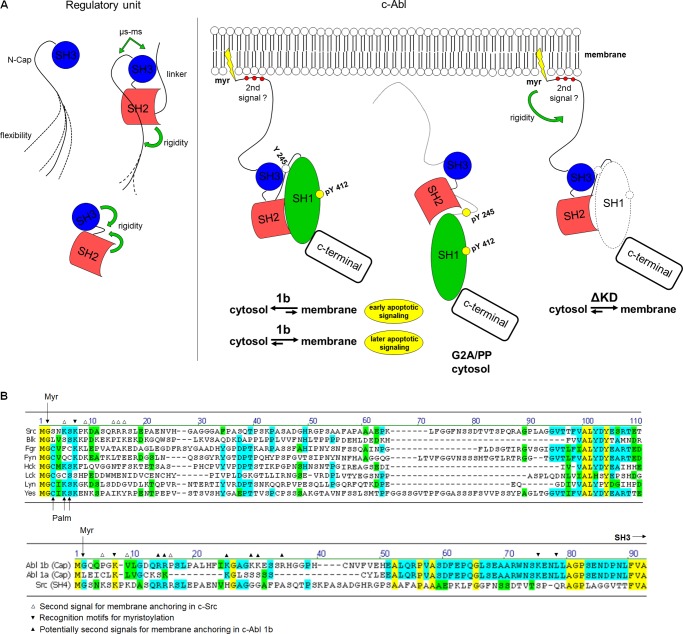
**Intramolecular couplings within the c-Abl regulatory unit.** *A*, a schematic representation summarizes the intramolecular dynamics for the isolated ABL-RD constructs and the full-length c-Abl constructs. *B*, N-Cap sequences from c-Src family kinase members, Abl-1b and Abl-1a, are aligned to identify the residues that are involved in the membrane anchoring of c-Src, the recognition motifs for myristoylation, and the potential residues involved in the membrane anchoring of Abl-1b.

SAXS and NMR are well designed approaches for assessing new insights on protein structure and function (31, 32). Changes in the dynamic properties and orientations of the N-Cap segment, such as those observed by our SAXS and NMR results, may arise upon c-Abl activation. Because myristoyl displacement is an important event in c-Abl activation and this modification is tethered via the N-Cap segment, the flexibility acquired from the uncoupled N-Cap-myristoyl tether would represent a potential condition to trigger different subcellular localization for the protein. A schematic representation in Fig. 8*A* summarizes the intramolecular couplings that may occur within the regulatory unit and the dynamic behavior of the N-Cap-myristoyl tether that targets the c-Abl protein to the membrane.

The N-Cap-myristoyl tether in the Abl-ΔKD is no longer constrained to associate within the C terminus of the kinase domain inside the cell. In this condition, the protein was translocated to the membrane of the cell, presumably to avoid the N-Cap unspecific proteolysis, as demonstrated by our GST-free ABL-RD and shorter constructs (Fig. 4, *A–G* and supplemental Table S1), and supports an additional stabilizing mechanism for this region. In this manner, N-Cap-SH2 interactions would not be sufficient to stabilize the entire N-terminal segment of the protein; thus, it is targeted to anchor in the membrane. Because we did not observe phosphorylated Tyr-245 in the whole cell extracts of the c-Abl-1b isoform, we assume that the N-Cap-SH2 interactions would occur in the membrane-bound protein; however, further investigation on membrane-enriched extracts would provide accurate confirmation. An extended interpretation of our results is that once the c-Abl anchors in the membrane, the N-Cap might adopt different orientations, such as those observed by the SAXS reconstructions from the GST fusion constructs.

In this manner, the partially compact organization of the N-Cap and the possibility for an N-Cap clamp orientation in the condition where the N terminus of the N-Cap is dynamically restricted (here determined by the GST fusion protein) suggest that the N-Cap region may also be intramolecularly coupled. This finding raises the question whether clamp orientations assumed for the N-Cap would be functionally important for the c-Abl protein during membrane anchoring and ultimately cell signaling.

Our SAXS data together with the Trp fluorescence assay supports the existence of synergistic stability between the N-Cap and the SH3 regions within the c-Abl regulatory unit. In the N-Cap-SH3 construct, the SH3 domain is partially unfolded, as observed by the average energy of Trp-118 and Trp-129 and the circular dichroism results, and one-third of the N-terminal segment of the N-Cap is disordered, as indicated by SAXS. Nevertheless, because SH2-L binds to the N-SH3 construct, the N-Cap region is partially stabilized by SH2 interactions, and the SH3 domain undergoes a local intermediate conformational change as observed by a 3-fold increase in the SH3 Trp fluorescence accompanied by the folding of this domain. Thus, the N-Cap and the SH3 domain participate synergistically upon SH2-L binding and demonstrate that they may respond as a unique segment within the protein.

In c-Abl, the acquisition of slow motion contributions in the N-Cap and SH3 domain upon binding of the N-Cap to the SH2 domain may represent a preferred state for SH3 signaling and raises the possibility that the SH3 domain would bind ligands even when the N-Cap interacts with the SH2 domain. Several c-Abl SH3-binding proteins have been identified and may act as candidates for an in *trans* c-Abl inhibition through SH3 binding (33–36).

During complete c-Abl activation, the movement of the SH3-SH2 domains to the top of the SH1 N-lobe (8) is likely accompanied by major changes in the intramolecular coupling within the regulatory unit. Alternatively, additional intramolecular mechanisms for the stability of the ABL-RD may occur in the active state, *e.g.* the interactions between the SH2 and the top of the SH1 N-lobe (8) and the anchoring of the protein to membrane regions via the N-Cap-myristoyl tether. The latter raises the question whether this functional N-Cap-myristoyl switch in c-Abl is just a *sine qua non* condition for protein regulation or whether c-Abl activation and myristoyl displacement are involved in additional functions inside the cell. We observed that the N-Cap-myristoyl tether may trigger the wild-type myristoylated c-Abl protein to anchor to the membrane, and this translocation to the plasma membrane was enhanced during the proapoptotic signaling of c-Abl. This observation links the participation of the N-Cap-myristoyl tether to a functional pathway triggered by the c-Abl kinase that should be explored in future studies. To note, transiently transfected wild-type c-Abl and variant forms Abl-PP, Abl-G2A, and Abl-G2A/PP were detected by immunoblots in crude membrane-enriched fractions obtained by differential velocity centrifugation (9), evidence that membrane targeting is a complex event and that additional mechanisms might be involved (37). In c-Src, myristoylation is important but not sufficient to anchor the protein to the membrane. A polybasic cluster of amino acids is required as a second signal for membrane anchoring (38). In the myristoylated c-Abl isoform (Abl-1b), the deleted 15–56 region of the N-Cap contains potential polybasic sites (Fig. 8*B*), which are absent in the shorter nonmyristoylated isoform. Therefore, this region might represent an additional element, together with the displaced myristoyl group, to direct the c-Abl kinase to the membrane. Further studies on the membrane-attached c-Abl will be required to solve the mechanistic of binding through the N-Cap-myristoyl switch and its requirement for c-Abl cellular function.

In conclusion, we provide new insights on the dynamic behavior for the extreme N-terminal segment of the c-Abl kinase. Our study, together with further exploration on the membrane-bound c-Abl environment, may offer a potential application to discover new targets for cancer therapy because the Bcr-Abl distribution in Bcr-Abl^+^ chronic myelogenous leukemia cells is cytosolic, and these cells are resistant to apoptotic events.

## Footnotes

2 The abbreviations used are:

SH: Src homology
N-Cap: N-terminal segment
SAXS: small angle x-ray scattering
SEC: size exclusion chromatography
RD: regulatory domain
KD: kinase-deficient
HSQC: heteronuclear single quantum correlation.

## References

[1] Taniguchi H. (1999) Protein myristoylation in protein-lipid and protein-protein interactions. Biophys. Chem. 82, 129–137 10631796 10.1016/s0301-4622(99)00112-x

[2] Boggon T. J. Eck M. J. (2004) Structure and regulation of Src family kinases. Oncogene 23, 7918–7927 15489910 10.1038/sj.onc.1208081

[3] Hantschel O. Superti-Furga G. (2004) Regulation of the c-Abl and Bcr-Abl tyrosine kinases. Nat. Rev. Mol. Cell Biol. 5, 33–44 14708008 10.1038/nrm1280

[4] de Klein A. van Kessel A. G. Grosveld G. Bartram C. R. Hagemeijer A. Bootsma D. Spurr N. K. Heisterkamp N. Groffen J. Stephenson J. R. (1982) A cellular oncogene is translocated to the Philadelphia chromosome in chronic myelocytic leukaemia. Nature 300, 765–767 6960256 10.1038/300765a0

[5] Heisterkamp N. Groffen J. (2002) Philadelphia-positive leukemia: a personal perspective. Oncogene 21, 8536–8540 12476299 10.1038/sj.onc.1206080

[6] Nowell P. Hungerford D. A. (1960) A minute chromosome in human chronic granulocytic leukemia. Science 132, 1497 10.1126/science.144.3623.1229 14150328

[7] Pluk H. Dorey K. Superti-Furga G. (2002) Autoinhibition of c-Abl. Cell 108, 247–259 11832214 10.1016/s0092-8674(02)00623-2

[8] Nagar B. Hantschel O. Seeliger M. Davies J. M. Weis W. I. Superti-Furga G. Kuriyan J. (2006) Organization of the SH3-SH2 unit in active and inactive forms of the c-Abl tyrosine kinase. Molecular Cell 21, 787–798 16543148 10.1016/j.molcel.2006.01.035

[9] Hantschel O. Nagar B. Guettler S. Kretzschmar J. Dorey K. Kuriyan J. Superti-Furga G. (2003) A myristoyl/phosphotyrosine switch regulates c-Abl. Cell 112, 845–857 12654250 10.1016/s0092-8674(03)00191-0

[10] Nagar B. Hantschel O. Young M. A. Scheffzek K. Veach D. Bornmann W. Clarkson B. Superti-Furga G. Kuriyan J. (2003) Structural basis for the autoinhibition of c-Abl tyrosine kinase. Cell 112, 859–871 12654251 10.1016/s0092-8674(03)00194-6

[11] Brasher B. B. Van Etten R. A. (2000) c-Abl has high intrinsic tyrosine kinase activity that is stimulated by mutation of the Src homology 3 domain and by autophosphorylation at two distinct regulatory tyrosines. J. Biol. Chem. 275, 35631–35637 10964922 10.1074/jbc.M005401200

[12] Cowan-Jacob S. W. Fendrich G. Manley P. W. Jahnke W. Fabbro D. Liebetanz J. Meyer T. (2005) The crystal structure of a c-Src complex in an active conformation suggests possible steps in c-Src activation. Structure 13, 861–871 15939018 10.1016/j.str.2005.03.012

[13] Patwardhan P. Resh M. D. (2010) Myristoylation and membrane binding regulate c-Src stability and kinase activity. Mol. Cell Biol. 30, 4094–4107 20584982 10.1128/MCB.00246-10 PMC2937550

[14] Druker B. J. Talpaz M. Resta D. J. Peng B. Buchdunger E. Ford J. M. Lydon N. B. Kantarjian H. Capdeville R. Ohno-Jones S. Sawyers C. L. (2001) Efficacy and safety of a specific inhibitor of the BCR-ABL tyrosine kinase in chronic myeloid leukemia. New Engl. J. Med. 344, 1031–1037 11287972 10.1056/NEJM200104053441401

[15] Ren R. (2005) Mechanisms of BCR-ABL in the pathogenesis of chronic myelogenous leukaemia. Nat. Rev. Cancer 5, 172–183 15719031 10.1038/nrc1567

[16] Quintás-Cardama A. Kantarjian H. Cortes J. (2007) Flying under the radar: the new wave of BCR-ABL inhibitors. Nat. Rev. Drug Discov. 6, 834–848 17853901 10.1038/nrd2324

[17] Hantschel O. Grebien F. Superti-Furga G. (2012) The growing arsenal of ATP-competitive and allosteric inhibitors of BCR-ABL. Cancer Res. 72, 4890–4895 23002203 10.1158/0008-5472.CAN-12-1276 PMC3517953

[18] Grebien F. Hantschel O. Wojcik J. Kaupe I. Kovacic B. Wyrzucki A. M. Gish G. D. Cerny-Reiterer S. Koide A. Beug H. Pawson T. Valent P. Koide S. Superti-Furga G. (2011) Targeting the SH2-kinase interface in Bcr-Abl inhibits leukemogenesis. Cell 147, 306–319 22000011 10.1016/j.cell.2011.08.046 PMC3202669

[19] Tugarinov V. Kanelis V. Kay L. E. (2006) Isotope labeling strategies for the study of high-molecular-weight proteins by solution NMR spectroscopy. Nat. Protoc. 1, 749–754 17406304 10.1038/nprot.2006.101

[20] de Oliveira G. A. Pereira E. G. Dias C. V. Souza T. L. Ferretti G. D. Cordeiro Y. Camillo L. R. Cascardo J. Almeida F. C. Valente A. P. Silva J. L. (2102) *Moniliophthora perniciosa* necrosis- and ethylene-inducing protein 2 (MpNep2) as a metastable dimer in solution: structural and functional implications. PLoS One 7, e45620 23029140 10.1371/journal.pone.0045620 PMC3454426

[21] Svergun D. I. (1992) Determination of the regularization parameter in indirect-transform methods using perceptual criteria. J. Appl. Crystallogr. 25, 495–503

[22] Svergun D. I. Petoukhov M. V. Koch. M. H. J. (2001) Determination of domain structure of proteins from X-ray solution scattering. Biophys. J. 80, 2946–2953 11371467 10.1016/S0006-3495(01)76260-1 PMC1301478

[23] Volkov V. V. Svergun D. I. (2003) Uniqueness of *ab initio* shape determination in small-angle scattering. J. Appl. Crystallogr. 36, 860–864 10.1107/S0021889809000338 PMC5023043 27630371

[24] Kozin M. B. Svergun D. I. (2001) Automated matching of high- and low-resolution structural models. J. Appl. Crystallogr. 34, 33–41

[25] Doniach S. (2001) Changes in biomolecular conformation seen by small angle X-ray scattering. Chem. Rev. 101, 1763–1778 11709998 10.1021/cr990071k

[26] Mertens H. D. T. Svergun D. (2010) Structural characterization of proteins and complexes using small-angle X-ray solution scattering. J. Struct. Biol. 172, 128–141 20558299 10.1016/j.jsb.2010.06.012

[27] Fushman D. Xu R. Cowburn D. (1999) Direct determination of changes of interdomain orientation on ligation: use of the orientational dependence of ^15^N NMR relaxation in Abl SH(32). Biochemistry 38, 10225–10230 10441115 10.1021/bi990897g

[28] Mantovani F. Piazza S. Gostissa M. Strano S. Zacchi P. Mantovani R. Blandino G. Del Sal G. (2004) Pin1 links the activities of c-Abl and p300 in regulating p73 function. Mol. Cell 14, 625–636 15175157 10.1016/j.molcel.2004.05.007

[29] Urist M. Prives C. (2004) The linchpin? Pin1 meets p73. Cancer Cell 5, 515–517 15193253 10.1016/j.ccr.2004.06.001

[30] Nam H. J. Haser W. G. Roberts T. M. Frederick C. A. (1996) Intramolecular interactions of the regulatory domains of the Bcr-Abl kinase reveal a novel control mechanism. Structure 4, 1105–1114 8805596 10.1016/s0969-2126(96)00116-5

[31] Lima L. M. Cordeiro Y. Tinoco L. W. Marques A. F. Oliveira C. L. Sampath S. Kodali R. Choi G. Foguel D. Torriani I. Caughey B. Silva J. L. (2006) Structural insights into the interaction between prion protein and nucleic acid. Biochemistry 45, 9180–9187 16866364 10.1021/bi060532d

[32] de Oliveira G. A. Rocha C. B. Marques M. de A. Cordeiro Y. Sorenson M. M. Foguel D. Silva J. L. Suarez M. C. (2013) Insights into the intramolecular coupling between the N- and C-domains of troponin C derived from high-pressure, fluorescence, nuclear magnetic resonance, and small-angle X-ray scattering studies. Biochemistry 52, 28–40 23215438 10.1021/bi301139d

[33] Shi Y. Alin K. Goff S. P. (1995) Abl-interactor-1, a novel SH3 protein binding to the carboxy-terminal portion of the Abl protein, suppresses v-*abl* transforming activity. Genes Dev. 9, 2583–2597 7590237 10.1101/gad.9.21.2583

[34] Dai Z. Pendergast A. M. (1995) Abi-2, a novel SH3-containing protein interacts with the c-Abl tyrosine kinase and modulates c-Abl transforming activity. Genes Dev. 9, 2569–2582 7590236 10.1101/gad.9.21.2569

[35] Zhu J. Shore S. K. (1996) c-ABL tyrosine kinase activity is regulated by association with a novel SH3-domain-binding protein. Mol. Cell Biol. 16, 7054–7062 8943360 10.1128/mcb.16.12.7054 PMC231708

[36] Wen S. T. Van Etten R. A. (1997) The PAG gene product, a stress-induced protein with antioxidant properties, is an Abl SH3-binding protein and a physiological inhibitor of c-Abl tyrosine kinase activity. Genes Dev. 11, 2456–2467 9334312 10.1101/gad.11.19.2456 PMC316562

[37] Resh M. D. (1999) Fatty acylation of proteins: new insights into membrane targeting of myristoylated and palmitoylated proteins. Biochem. Biophys. Acta 1451, 1–16 10446384 10.1016/s0167-4889(99)00075-0

[38] McLaughlin S. Aderem A. (1995) The myristoyl-electrostatic switch: a modulator of reversible protein-membrane interactions. Trends Biochem. Sci. 20, 272–276 7667880 10.1016/s0968-0004(00)89042-8

